# *RAS* Mutational Status Detection in Tissue, Plasma, and Stool Samples for Colorectal Cancer

**DOI:** 10.1155/2020/5419634

**Published:** 2020-04-11

**Authors:** Liuying Zhu, Yuanhe Wang, Yang Zhou, Qian Dong, Yunpeng Liu, Jingdong Zhang

**Affiliations:** ^1^Medical Oncology Department of Gastrointestinal Tumors, Liaoning Cancer Hospital & Institute, Cancer Hospital of China Medical University, No. 44, Xiaohe Road, Dadong District, Shenyang, 110042 Liaoning Province, China; ^2^Department of Medical Oncology, The First Hospital of China Medical University, Shenyang, China; ^3^Key Laboratory of Anticancer Drugs and Biotherapy of Liaoning Province, The First Hospital of China Medical University, Shenyang, China

## Abstract

**Objective:**

*RAS* gene testing on tumor tissue biopsies is required for metastatic colorectal cancer (CRC) patients. However, it is infeasible for patients after curative surgery and repeated biopsy. This study is aimed at evaluating the consistency of *RAS* genes in patient's plasma, stool, and tumor tissue samples, to explore whether plasma and stool samples can supplement or replace tumor tissue to assess baseline *RAS* gene status.

**Methods:**

Between June 2016 and October 2017, 53 patients with stage I-IV CRC from the Liaoning Cancer Hospital and the Department of Medical Oncology of the First Hospital of China Medical University were enrolled in the study. Patient tissues, peripheral blood, and stool samples were collected, and *RAS* gene tests were performed.

**Results:**

Analysis of the *KRAS* gene in tissue, plasma, and stool samples from 53 CRC patients detected 25 cases (47%) of *KRAS* gene mutations in the tissue samples, 20 cases (38%) of *KRAS* gene mutations in plasma, and 18 (34%) *KRAS* gene mutations in fecal samples. The overall consistency of *KRAS* gene status between tissue samples and plasma samples was 77.4% (*p* ≤ 0.05) and between tissue samples and stool samples was 83% (*p* ≤ 0.05). In stage IV cases, the agreement of *KRAS* gene status between tissue and plasma samples was 93.8% (*p* ≤ 0.05) and 93.8% (*p* ≤ 0.05) between tissue and stool samples.

**Conclusion:**

There was a high overall consistency in *KRAS* mutational assessment between plasma, stool, and tissue samples. In stage IV patients, the consistency of *KRAS* gene detection between tissue and stools or plasma was higher.

## 1. Introduction

Nearly one million new cases of colorectal cancer (CRC) are diagnosed worldwide each year [1, 2], and it is one of the leading causes of cancer-related death [3–5]. However, in recent years, due to improvements in early detection and comprehensive treatment, the median overall survival (OS) has reached 30 months or longer [6–9].

Only CRC patients with wild-type *RAS* have benefited from anti-Epidermal Growth Factor Receptor (EGFR) treatment. As such, rapid and accurate detection of *RAS* gene mutations is vital for personalized CRC treatment. The use of tumor tissue to detect the *RAS* gene is the current gold standard in clinical practice and is also a frequently used detection indicator in clinical practice [10, 11].

The detection of circulating tumor DNA (ctDNA) is a minimally invasive detection method, which has obvious advantages over traditional tissue detection and is also more representative of tumor heterogeneity. ctDNA is derived from tumor cells, and the false positive detection for ctDNA is lower than that of protein biomarkers [12, 13]. It has been reported that the average half-life of fetal DNA is 16.3 minutes (with range 4-30 minutes) and that it is effectively eliminated within 2 hours [14]. Similarly, the half-life of ctDNA is also short [15, 16] and it allows us to monitor the dynamics of the tumor in units of hours, instead of weeks or even months, unlike tissue biopsies [17].

In recent years, noninvasive multitarget fecal DNA (mt-sDNA) detection technology has gradually matured. Tumor cells shed on the surface of precancerous lesions and are released into the feces. Through direct histological observation, it is found that shedding of tumor cells is a continuous process and is more frequent than that of ordinary epithelial cells. This may be due to excessive proliferation, which reduces cell-to-cell or cell-to-basement membrane adhesion [18]. This consistent tumor cell shedding allows us to monitor tumor-derived fecal DNA in a single stool sample. mt-sDNA testing can be used to detect 11 biomarkers (seven of which are DNA mutations in the *KRAS* gene) in stool samples, and there is a high consistency in results between fecal testing and tissue testing [19–21]. This study is aimed at investigating the consistency of *RAS* gene detection in plasma, stool, and tumor tissue in patients with CRC.

## 2. Material and Methods

### 2.1. Study Design

In this study, we compared the consistency of *RAS* gene detection in plasma, stool, and tumor tissue in patients with CRC. All participants were enrolled between June 2016 and October 2017 in the Liaoning Cancer Hospital and the Department of Medical Oncology of the First Hospital of China Medical University. Informed written consent was obtained from all patients enrolled in this study. This study was approved by the Liaoning Cancer Hospital Institutional Review Board.

#### 2.1.1. Diagnosis and Inclusion Criteria

Patients recruited met the following criteria: diagnosed with CRC by histopathology, aged between 18 and 80 years old, agreed to conduct histological *RAS* gene testing, were willing to provide blood and stool samples in a tumor-bearing state, never received anti-EGFR therapy, and signed informed consent.

### 2.2. Clinical Procedures

Patient peripheral blood and stool samples were collected from the remainder of routine admission examination samples. Peripheral blood samples of 5 ml per patient were taken, placed in a blood collection tube supplemented with ethylenediaminetetraacetic acid, and sent to the laboratory within 4 hours. Here, samples were centrifuged at 3000 rpm at 4°C, and the plasma supernatant was collected and sealed in a low-adsorption EP tube for cryopreservation at -80°C. Stool samples (preferably dry) of 1 g were sealed in cryotubes and sent to the laboratory within 2 hours for cryopreservation at -80°C.

Amongst the 53 patients enrolled in this study, 49 patients were naive, while four had systemic treatment (2 with chemotherapy plus bevacizumab and 2 with chemotherapy alone).

### 2.3. Polymerase Chain Reaction (PCR)

We used the QIAamp DNA Mini Kits (QIAGEN 51306) and the QIAamp DNA Stool Mini Kit (QIAGEN 51504) to extract DNA from the peripheral blood and stool samples, respectively. The DNA quality was considered sufficient when sample DNA concentration ≥ 5 ng/*μ*l and OD260/OD280 = 1.4–2.0. The Typer software was used to interpret the molecular weight peaks detected by mass spectrometry, which were transformed to show the molecular weight peaks corresponding to single-nucleotide polymorphism sites.

Genetic testing of histology samples was performed using the standard procedures validated by each hospital, and the reported data were used for this investigation.

### 2.4. Statistical Analysis

Statistical analyses for paired sample detection were performed using SPSS software (SPSS version 25, SPSS Inc.).

Groups and formulae for analysis were specified as follows (Table 1): *KRAS* mutations in tissue and plasma or stool, A; *KRAS* mutations in plasma or stool but not tissue, B; *KRAS* mutations in tissue but not in plasma or stool, C; and no *KRAS* mutations in tissue and plasma or stool, D. Positive percentage agreement (PPA) = A/(A + C); negative percentage agreement (NPA) = D/(B + D); overall agreement = (A + D)/(A + B + C + D).

## 3. Results

### 3.1. Patient Characteristics and Gene Test Results

Between June 2016 and October 2017, fifty-three CRC patients were enrolled, including 37 males (69.8%) and 16 females (30.2%). There were 46 patients (86.8%) with the primary tumor located in the left side colorectum and seven patients (13.2%) with the primary tumor in the right side colon. For the purpose of this study, the colon is divided into the left side colorectum and right side colon at the splenic flexure. Patient tumor stages were as follows: seven (13.2%) stage I, 13 (24.5%) stage II, 17 (32%) stage III, and 16 (30.3%) with stage IV. At the time of sample collection, no patients had received anti-EGFR-targeted therapy, 49 (92.4%) patients had not received treatment, and four (7.6%) patients with metastatic CRC had received therapy (two of these were chemotherapy plus bevacizumab and the others were chemotherapy alone). Patient parameters are summarized in Table 2.

The median concentration of plasma ctDNA was 8 ng/*μ*l. There was no significant correlation between plasma DNA concentration and the level of carcinoembryonic antigen (*r* = 0.2266). The median concentration of DNA extracted from feces was 62 ng/*μ*l. The stool DNA concentration was moderately correlated with the value of carcinoembryonic antigen (*r* = 0.6721).

A total of 29 mutation sites were tested for and are shown in Table 3.  Table 4 shows the total *RAS* mutations detected in patient samples. Of the 53 patients, 25 patients (47%) had *KRAS* gene mutations detected in tumor tissue, 19 patients (35.8%) from plasma, and 19 patients (35.8%) from stool. No *NRAS* mutations were detected from tissue, plasma, and fecal samples. *BRAF* gene mutations were detected in tissue and plasma from six patients (11.3%) and in stool from three patients (5.7%).

## 4. Patient Characteristics and the Agreement of *KRAS* Gene Mutation Detection in Plasma versus Tissue

For the 25 patients in whose tissue sample *KRAS* mutation was detected, 16 also had a *KRAS* mutation detected in plasma (PPA of 64%) and 18 in stool (PPA of 72%). Amongst the 28 patients determined to be *KRAS* wild type (wt) from tissue analysis, 25 also had *KRAS* wt in plasma (NPA of 89.3%) and 27 also had *KRAS* wt in stool samples (NPA of 96.4%). The overall agreement between *KRAS* status between plasma and tissue samples was 77.4% (41/53 patients, *p* ≤ 0.05) and between stool and tissue samples was 84.9% (45 of 53 patients, *p* ≤ 0.05). These results are shown in Table 5.

A combined blood and stool metric (plasma+stool), considered positive if a *RAS* mutation is detected in either, improves the PPA with tissue samples. Here, the PPA was 96% (24/25 patients), the NPA was 85.7% (24/28 patients), and the overall agreement rate was 48 in 53 patients (90.6%, *p* ≤ 0.05). These results are shown in Table 6.

## 5. *KRAS* Mutation Detection in Patients Separated by Tumor Stage

We investigated the agreement between *KRAS* status of plasma and tissue samples, according to the clinical stage of the cancer. Stages I, II, III, and IV gave overall agreements of 28.6% (2/7, *p* > 0.05), 69.2% (9/13, *p* > 0.05), 88.2% (15/17, *p* ≤ 0.05), and 93.8% (15/16, *p* ≤ 0.05). The same analysis for stool and tissue samples gave overall agreements of 57.1% (4/7, *p* > 0.05), 84.6% (11/13, *p* > 0.05), 88.2% (15/17, *p* ≤ 0.05), and 93.8% (15/16, *p* ≤ 0.05) for stages I, II, III, and IV, respectively. These results are summarized in Table 7.

## 6. Comparison of *KRAS* Mutation Status in Blood, Stool, and Tissue Samples from Primary Tumors of the Left Side Colorectum and Right Side Colon

In the study population, there were 46 patients with left-sided CRC and seven patients with right-sided CRC. The overall agreement between plasma and tissue samples was similar in left- and right-sided CRCs (73.9% and 71.4%, respectively). However, while the overall agreement between fecal and tissue sample *KRAS* detection was 91.3% (42/46) in left-sided CRC, it was just 42.9% (3/7) in right-sided CRC. These results are shown in Table 8.

## 7. Consistency of BRAF Gene Detection in Patient Samples

In six patients, BRAF mutation was detected in tissue samples. In four of these patients, BRAF mutation was also detectable in plasma (PPA of 66.7%) and four had a BRAF mutation in stool (PPA of 66.7%). Of the 47 patients with wild-type BRAF detected in tissue samples, 46 also had wild-type BRAF detected in ctDNA (NPA of 97.5%) and 47 also had a BRAF wt (NPA of 100%). The overall agreement of *BRAF* mutational status between plasma and tissue samples was 94.3% (50/53 patients, *p* ≤ 0.05) and between stool and tissue samples was 96.2% (51/53 patients, *p* ≤ 0.05). These findings are shown in Tables 9 and 10 for plasma and stool comparisons, respectively.

## 8. Discussion

As only CRC patients with wild-type *RAS* genes benefit from anti-EGFR treatment, it is important to detect the *RAS* gene accurately and conveniently. Gene detection from tumor biopsy tissue is the gold standard, but repeating tissue biopsy by enteroscopy may increase related complications [22, 23] and only reflect the state of the genome at a certain site and time. Many patients have distant metastases at the time of diagnosis, and tissue biopsy specimens cannot be obtained by surgery in these cases [24, 25]. Here, we investigate whether plasma and stool samples can be used as a supplement or alternative to tumor tissue for *RAS* gene status testing.

As early as 1992, Sidransky et al. published an article in Science, which reported that the agreement rate of *KRAS* mutation in feces and tumor tissues was 89% (8/9) [26]. Similarly, a study by Vidal et al. showed that the overall agreement of *RAS* mutation status between ctDNA and tissue samples was 93% (107/115), with a positive agreement rate of 96.4% and a negative agreement rate of 90% [27]. In this study, plasma, fecal, and tissue samples were simultaneously included in comparative analysis. The high agreement between tissue and fecal or plasma *KRAS* gene assays supports the use of peripheral blood or feces as a viable complement or alternative to tissue DNA testing. The results also showed that combining *KRAS* gene detection results from plasma and stool samples increased the positive rate of detection (96%, *p* ≤ 0.05), indicating that these two metrics can complement each other.

When tumor cells are shed, or once cells become apoptotic, small fragments of DNA are released into the circulatory system. Mutations in this ctDNA have been detected in almost all types of cancer, and the later the tumor stage or the higher the malignancy of the tumor, the higher the frequency of mutations detected by ctDNA. The same is true for DNA testing in stool samples. Anti-EGFR treatment may have some impact on the status of the RAS gene; at the time of sample collection, all patients had not received anti-EGFR-targeted therapy. 49 (92.4%) patients had not received antineoplaston, 4 (7.6%) patients with mCRC had received therapy (2 of them were chemotherapy+bevacizumab and the others were chemotherapy alone). With tumor remission after treatment, gene abundance in plasma DNA is affected. The gene abundance of RAS wild type is relatively sensitive; it may decrease below the detection line after chemotherapy, making it undetectable; and the RAS wild type may also change to RAS mutant type after treatment. However, in this study, the 4 treated patients had the same RAS gene status. In this study, patients were grouped according to AJCC 8.0 cancer stages and analyzed separately. The agreement between sample mutation status readouts was higher in patients with stages IV and III than in patients with stages I and II. However, the agreement between feces and tissue in stages I and II was significantly higher than that in plasma and tissue (57.1% to 28.6% in stage I and 84.6% to 69.2% in stage II, for feces and plasma, respectively). In colonic tumors, whether it is a malignant tumor or an advanced adenoma, exfoliated cells detached from their surface can be released into the feces directly. Therefore, in early cancer stages, tumor DNA in stool samples may be easier to detect than ctDNA and may be a better choice than blood sample analysis for early screening.

It has been reported that mt-sDNA testing has a similar consistency rate for left- and right-sided CRCs [28]. The right side colon has a large intestinal lumen and a thin intestinal wall and expands easily. Its physiological function is to absorb water, electrolytes, and some glucose. As such, the contents of the right-sided colon are mostly liquid or semiliquid, which affects the purity of tumor DNA extraction in the feces. In contrast, the left-sided colorectum has a narrow intestinal lumen and its main physiological function is to absorb water and store stools. Therefore, the contents of the left-sided colorectum are relatively dry, which are conducive to tumor DNA extraction and gene detection. The data here show that blood test results are not affected by the location of the primary tumor site (with left side colorectum agreement with tissue status at 73.9% and right side colon at 71.4%) but that fecal testing performs better in the left side colorectum (91.3% agreement compared with 42.9% in the right side colon).

The sample size in this study is relatively small; however, larger studies are required to validate these results.

## 9. Conclusion

The high agreement between plasma-, fecal-, and tissue-based detection of *RAS* mutational status supports the use of plasma and fecal *RAS* gene detection as a viable alternative to tissue detection in CRC patients. The detection accuracy from plasma and fecal samples is higher for later stage CRCs, while for early stage screening, analysis of fecal samples may be a better choice.

## Figures and Tables

**Table 1:** 

	Tissue *KRAS* status			
Plasma/stool KARS status		Mutated	No mutated	
	Mutated	A	B	
	No mutated	C	D	

**Table 2:** Patient characteristics (*n* = 53).

Characteristics	Cases (*n*)	Percentage (%)
Gender		
Male	37	69.8
Female	16	30.2
Tumor site		
Left side colorectum	46	86.8
Right side colon	7	13.2
Clinical stage AJCC 8.0		
I	7	13.2
II	13	24.5
III	17	32
IV	16	30.3
Distant metastatic sites		
1	6	37.5
2	5	31.2
≥3	5	31.2
Treatment before sampling		
Untreated	49	92.4
Treated	4	7.6

**Table 3:** Gene and mutation sites.

Test gene	Codon	Mutation
*KRAS*	*KRAS*-Exon2	G12S, G12D
	*KRAS*-Exon2	G12C, G12R, G12V, G12A, G13C
	*KRAS*-Exon2	G13D
	*KRAS*-Exon3	Q61L, Q61R, Q61H
	*KRAS*-Exon4	K117N, A146T, A146V, A146P

*NRAS*	*NRAS*-Exon2	G12D, G12S
	*NRAS*-Exon2	G13D
	*NRAS*-Exon2	G13R, G12C, G12V, G12A, G13V
	*NRAS*-Exon3	Q61R, Q61K, Q61L, Q61H
	*NRAS*-Exon4	A146T

*BRAF*	*BRAF*-Exon15	V600E

**Table 4:** Gene mutation rates.

	Tissue (%)	Plasma (%)	Stool (%)
*KRAS* mutations	25 (47)	19 (35.8)	19 (35.8)
*BRAF* mutations	6 (11.3)	6 (11.3)	3 (5.7)

**Table 5:** Agreement of *KRAS* status.

Concordance	PPA (%)	NPA (%)	Overall agreement (%)	*p*
Plasma & tissue	64 (16/25)	89.3 (25/28)	77.4 (41/53)	*p* ≤ 0.05
Stool & tissue	72 (18/25)	96.4 (27/28)	84.9 (45/53)	*p* ≤ 0.05

**Table 6:** The agreement of *KRAS* gene status between plasma combined with stool and tissue samples. Data presented as patient numbers.

	Tissue			
		Mutated	Unmutated	Total
Plasma+stool	Mutated	24	4	28
	Unmutated	1	24	25
	Total	25	28	53

**Table 7:** The agreement of mutant *RAS* detection from plasma, stool, and tissue samples in stage I to IV patients.

Stage	PPA		NPA		Overall agreement	
	Plasma & tissue	Stool & tissue	Plasma & tissue	Stool & tissue	Plasma & tissue	Stool & tissue
I	33.3% (2/6)	50% (3/6)	0% (0/1)	100% (1/1)	28.6% (2/7, *p* > 0.05)	57.1% (4/7, *p* > 0.05)
II	66.7% (4/6)	85.7% (6/7)	71.4% (5/7)	85.7% (6/7)	69.2% (9/13, *p* > 0.05)	84.6% (11/13, *p* > 0.05)
III	75% (6/8)	75% (6/8)	100% (9/9)	100% (9/9)	88.2% (15/17, *p* ≤ 0.05)	88.2% (15/17, *p* ≤ 0.05)
IV	80% (4/5)	80% (4/5)	100% (11/11)	100% (11/11)	93.8% (15/16, *p* ≤ 0.05)	93.8% (15/16, *p* ≤ 0.05)

**Table 8:** The agreement between *KRAS* mutation status from plasma or stool and tissue samples, based on the sidedness of CRC.

Site	PPA		NPA		Overall agreement	
	Plasma & tissue	Stool & tissue	Plasma & tissue	Stool & tissue	Plasma & tissue	Stool & tissue
Left side colorectum	65% (13/20)	85% (17/20)	88.5% (23/26)	96.2% (25/26)	73.9% (36/46, *p* ≤ 0.05)	91.3% (42/46, *p* ≤ 0.05)
Right side colon	60% (3/5)	20% (1/5)	100% (2/2)	100% (2/2)	71.4% (5/7, *p* > 0.05)	42.9% (3/7, *p* > 0.05)

**Table 9:** Agreement in detection rates between plasma and tissue samples for *BRAF*.

	Tissue *BRAF* status			
Plasma *BRAF* status		Mutated	Unmutated	Total
	Mutated	4	1	5
	Unmutated	2	46	48
	Total	6	47	53

**Table 10:** Agreement in detection rates between plasma and stool samples for *BRAF*.

	Tissue *BRAF* status			
Stool *BRAF* status		Mutated	Unmutated	Total
	Mutated	4	0	4
	Unmutated	2	47	49
	Total	6	47	53

## Data Availability

The data used to support the findings of this study are included within the article.
